# Testing the Validity of the Montgomery–Koyama–Smith Equation for Calculating the Total Petal Area per Flower Using Two Rosaceae Species

**DOI:** 10.3390/plants13243499

**Published:** 2024-12-15

**Authors:** Chuanlong Zhao, Jinfeng Wang, Youying Mu, Weihao Yao, Hui Wang, Peijian Shi

**Affiliations:** 1 Co-Innovation Center for Sustainable Forestry in Southern China, Bamboo Research Institute, Nanjing Forestry University, #159 Longpan Road, Nanjing 210037, China; zhaochl@njfu.edu.cn (C.Z.); jfwang@njfu.edu.cn (J.W.); muyouying@njfu.edu.cn (Y.M.); whyao@njfu.edu.cn (W.Y.); pjshi@njfu.edu.cn (P.S.); 2 College of Landscape Architecture, Nanjing Forestry University, #159 Longpan Road, Nanjing 210037, China

**Keywords:** Montgomery equation, Montgomery–Koyama–Smith equation, Rosaceae, individual petal area, total petal area per flower

## Abstract

The size of floral organs is closely related to the successful reproduction of plants, and corolla size is, to some extent, indicative of the size of floral organs. Petals are considered to be homologous to leaves, so we also attempted to estimate the area of a single petal using the method that is typically employed for estimating single leaf area (i.e., the Montgomery equation). Additionally, we estimated the total petal area per flower (*A*_T_; i.e., the whole corolla area) using the method designed for estimating the total leaf area per shoot (i.e., the Montgomery–Koyama–Smith equation). The Montgomery equation (ME) estimates the leaf area by assuming that the leaf area is proportional to the product of leaf length and width. The Montgomery–Koyama–Smith equation (MKSE) assumes that the total leaf area per shoot is proportional to the product of the sum of individual leaf widths and the maximum individual leaf length. To test the validity of the ME for predicting petal area, a total of 1005 petals from 123 flowers of two Rosaceae species, which exhibit a certain variation in petal shape, were used to fit the relationship between the petal area (*A*) and the product of petal length (*L*) and width (*W*). Two equations, including the MKSE and a power-law equation (PLE), were used to describe the relationship between the total petal area per flower and the product of the sum of individual petal widths and the maximum individual petal length. The root-mean-square error (RMSE) and the Akaike information criterion (AIC) were used to measure the goodness of fit and the trade-off between the goodness of fit and model’s structural complexity for each equation. The results show that the ME has a low RMSE value and a high correlation coefficient when fitting the relationship between *A* and *LW* for either of the two species. Additionally, the MKSE and the PLE exhibit low RMSEs and AICs for estimating the *A*_T_ of both Rosaceae species. These results indicate that the ME, MKSE, and PLE are effective in predicting individual petal area and total corolla area, respectively.

## 1. Introduction

Flowers are the sexual reproductive organs of angiosperms, and their components are considered metamorphic leaves. Among them, petals, as part of the floral organs, were noted as metamorphic leaves by J. W. von Goethe in his writings as early as the 18th century, a viewpoint that has since been confirmed by genetic studies [1,2]. For example, in the *Arabidopsis* ABC triple mutant—*apetala2*, *pistillata*, and *agamous1* [3,4], and in the quadruple mutant—*ap1*, *ap2*, *ap3/pi*, and *ag* [5], the absence of floral organ-identifying activities causes all floral organs to develop as leaves. Unlike leaves, petals generally do not have the ability to photosynthesize, but they play an important role in the reproduction of plants [6,7]. For example, petals are often used as visual attractants/guides, nectar producers/preservers/coverings, and/or landing platforms for pollinators [8,9,10]. Kaczorowski et al. [11] found that the differences in corolla shape and size both can influence the feeding and pollination behavior of *Manduca sexta*; however, corolla size may be more important for feeding and pollination preference by *M. sexta* than corolla shape.

Leaf area is often used as a measure of leaf size and as a serial homologue of leaves. In a similar way, petal area is also considered a measure of petal size. Currently, leaf area can be determined through either direct or indirect methods. Direct methods for measuring leaf area, such as blueprinting, paper weighing, grid counting, and using a leaf area meter, are accurate but destructive and time-consuming [12,13]. Several indirect methods are available for estimating leaf area, including computer vision, image processing techniques, and simple linear regression models [14,15,16]. Among them, computer vision and image processing techniques have found extensive application across various domains, including leaf area determination. For instance, they are used for outlining leaf geometry by marking specific landmarks along the lamina boundary and forming a polygon for leaf area computation, as well as scanning leaves using photo scanners and utilizing image processing software, e.g., ImageJ (version 2.3.0) [16,17]. Although this method is accurate, it requires the leaf to be very flat and has some limitations, such as the need for destructive sampling, as well as scanning or photographing intact leaves limits large-scale application. Simple regression models are based on the linear relationships between leaf one-dimensional variables (e.g., leaf length and width) and the leaf area. The most commonly used simple regression model is the Montgomery equation (ME), which assumes that the leaf area is proportional to the product of leaf length and width, and it has proven to be an effective tool for nondestructively estimating the lamina area [16,17,18,19,20]. In addition, the ME has been shown to effectively estimate the area of non-planar leaves, e.g., the leaves of *Ilex cornuta* [21], as well as leaves with intricate shapes [19,20,22]. Wang et al. [23] demonstrated the validity of the ME in estimating tepal (petals and petal-like floral parts) areas in *Magnolia* × *soulangeana*. However, its applicability to petals of Rosaceae species with irregular shapes remains unexplored. Once the ME is confirmed to be effective in predicting petal area, it will simplify the experimental process, save time and labor, and then avoid the high cost of directly measuring the petal area. This is especially important for research involving large-scale plant samples. Additionally, the fitting parameters between leaf/petal area and the product of length and width for different species are important for understanding their phylogenetic relationships. For example, Wang et al. [23] found that the ME-fitted parameters for tepals and leaves were very similar in *M*. × *soulangeana*. This provides metrological evidence of floral and foliar homology in plants.

Petals form a corolla, and corolla size is often used to characterize flower size. Thus, it is also important to study corolla size [24,25]. Koyama and Smith [26] recently proposed a method for calculating the total leaf area per shoot (denoted as *A*_shoot_). They defined the sum of all individual leaf widths per shoot as the “foliage length” (*L*_f_) of the shoot, and the maximum individual leaf length per shoot as the “foliage width” (*W*_f_) of the shoot. *A*_shoot_ was hypothesized to be proportional to *L*_f_*W*_f_. Although the method has been validated for calculating the total leaf area per shoot in several plant species [26,27], its validity has not been tested for calculating the sum of the areas of other plant organs, tissues, and cells in a given statistical unit, e.g., the total petal area in a flower and the total stomatal area in a micrograph. In fact, flowers can be regarded as a modified branch, with their floral organs being homologous to foliage leaves [1,2]. This concept of taxonomic homology between flowers and leaves motivated us to conduct the present study. For convenience, we refer to the equation proposed by Koyama and Smith [26] as the Montgomery–Koyama–Smith equation (MKSE).

In this study, we selected two Rosaceae species for our experiment, i.e., *Malus halliana* var. *Parkmanii* and *Prunus × kanzakura* cv. *Kawazu-zakura*. Both of them are important ornamental species [28,29], and the petal shapes of these two species are distinctly different, providing a robust basis for validating the ME and the MKSE. We sampled flowers in March 2024 and obtained the petal area, length, and width data. The objectives of this study were to (1) evaluate the validity of the ME in estimating individual petal area, (2) test the accuracy of the MKSE in predicting the total petal area per flower, and (3) explore alternative equations that may outperform the MKSE in these estimations.

## 2. Materials and Methods

### 2.1. Species and Flower Collection Information

Two Rosaceae species, i.e., *Malus halliana* var. *Parkmanii* (denoted as Mh for simplicity) and *Prunus × kanzakura* cv. *Kawazu-zakura* (denoted as Pk for simplicity) grown at the Nanjing Forestry University Xinzhuang Campus were used. The studied Mh is a semi-double variety of deciduous small trees in the genus *Malus* that is native to China. It flowers profusely every year, and the flowers, which droop on slender pedicels, are rose red with obovate petals. Owing to its small size, Mh has been widely used for landscape arrangement in small gardens [28]. The studied Pk is a cultivar of deciduous trees in the genus *Prunus* and is thought to be a hybrid between *Prunus lannesiana* var. *speciosa* and *Prunus campanulata* [29]. Its flowers are pink with five broadly ovate to rounded petals, each having a concave apex, which bloom in early spring; this plant is an important ornamental species. These two species belong to different genera (*Malus* and *Prunus*) within the same family (Rosaceae) and exhibit significant differences in petal shapes, offering a diversified perspective and helping to verify the applicability and reliability of the proposed method across different plant groups. Furthermore, both species are important ornamental plants that are widely used in horticulture and landscape design, adding practical application value to this study.

In March 2024, we collected 60 flowers of Mh and 63 flowers of Pk at the Nanjing Forestry University campus in Nanjing, Jiangsu Province, China (118°48′33″ E, 32°4′48″ N). As a rule of thumb, a sample size greater than 30 is considered large and sufficient for statistical analysis. Although a larger sample size is generally preferred in practice, we chose to collect approximately 60 samples in this study to minimize the impact of destructive samplings on the growth of plants. As soon as the flowers were picked, they were placed in a foam box (54 × 39.5 × 30 cm) with ice packs and transported back to the laboratory within ten minutes while being stored in a car refrigerator (Meiling ML-C2-24S, Hefei, China). To avoid direct contact between the flowers and the ice packs, we used a layer of cardboard as a separator. The ice packs served to maintain a low-temperature environment. Additionally, we made efforts to minimize contact between the flowers and ensured a smooth transportation process to prevent any damage to them.

### 2.2. Scanning and Processing of Petal Images

**Figure 1:**
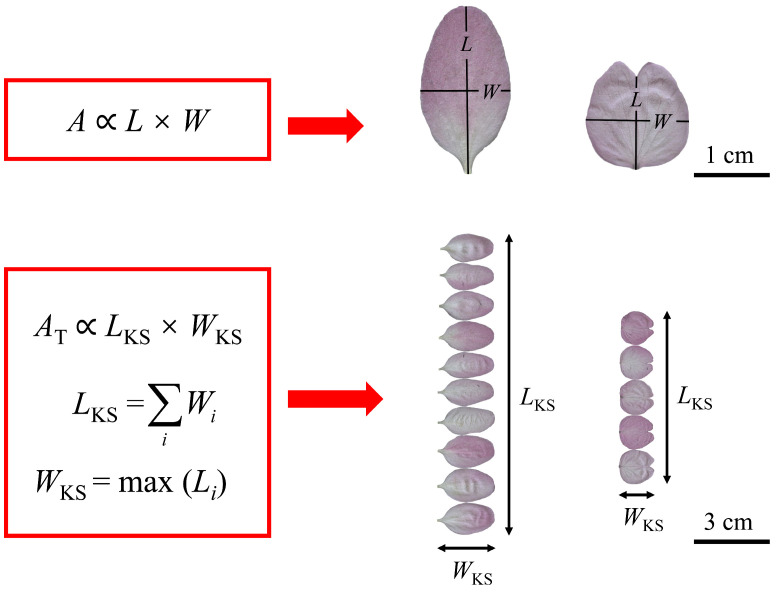
An illustration of the Montgomery equation and the Montgomery–Koyama–Smith equation with different variables for *Malus halliana* var. *Parkmanii* (on the second column) and *Prunus × kanzakura* cv. *Kawazu-zakura* (on the third column). Here, *A* is the individual petal area; *L* and *W* are the individual petal length and width, respectively; *A*_T_ is the total petal area per flower; *L*_KS_ is the sum of the petal widths per flower; and *W*_KS_ is the maximum petal length per flower.

We carefully separated the petals from each flower, scanned them using a photo scanner (V550, Epson Indonesia, Batam, Indonesia), and saved them in the .jpg format at 600 dpi resolution. Then, Adobe Photoshop 2021 (version 22.4.2; Adobe Systems Incorporated, San Jose, CA, USA) was used to obtain black-and-white images of the petal boundaries that were saved as bitmap images at 600 dpi resolution. A Matlab (version ≥ 2009a; MathWorks, Natick, MA, USA) procedure developed by Shi et al. [30] and Su et al. [31] was used to obtain the planar coordinates of each petal boundary by counting the pixel values of each image. We used the “bilat” function in the “biogeom” package (version 1.3.5; Shi et al.) [32] based on the R software (version 4.3.2; R Core Team, 2023) [33] to calculate the petal area, length, and width (see Figure 1 for the definition of the length and width of the petals of the two species). Based on Koyama and Smith’s method [26] for calculating the total leaf area in a shoot, we separated all the petals from the flower’s receptacle and placed them side by side on a flat plane, as shown in Figure 1. The total petal area per flower (denoted as *A*_T_), the sum of individual petal widths (denoted as *L*_KS_), and the maximum individual petal length (denoted as *W*_KS_) were calculated according to the definitions of Koyama and Smith [26], as illustrated in Figure 1. Here, the subscript “KS” is the acronym of the family names of Koyama and Smith.

The size measurements for each petal are tabulated in the online Table S1.

### 2.3. Statistical Methods

Tukey’s honestly significant difference (HSD) test with a 0.05 significance level [34] was used to determine whether there were significant differences in the means of petal area (*A*), petal length (*L*), petal width (*W*), the ratio of petal width and length (*W*/*L*), the total petal area per flower (*A*_T_), and the ratio of the maximum petal length (*W*_KS_) per flower to the sum of the petal widths (*L*_KS_) per flower between the two species.

Montgomery [18] proposed a proportional relationship between the leaf area and the product of leaf length and width; the equation came to be known as the Montgomery equation (ME), and its validity has been verified for the leaves of broad-leaved monocot and eudicot species [16,17,18,19,20]. The ME was used to test whether the empirically determined petal area (*A*) had a proportional relationship with the petal length (*L*) and width (*W*), i.e.,

(1) A=k×LW,

where *k* is the proportionality coefficient (denoted as the Montgomery parameter) to be estimated. To stabilize the variance of petal area, the two sides of Equation (1) were log-transformed:

(2) $$\log\left(A\right)=a+\log\left(LW\right),$$

where the parameter *a* (= log(*k*)) is estimated using ordinary least squares regression protocols to fit Equation (2). The predicted value of petal area ($\hat{A}$) is calculated by the following formula:

(3) $$\hat{A}=\hat{k}\times LW,$$

where $\hat{k}$ is the estimated value of the Montgomery parameter. The-root-mean square error (RMSE) was then used to assess the goodness of fit of the linear regression:

(4) $$\mathrm{RMSE}=\sqrt{\frac{1}{n_{2}}{\sum_{i=1}^{n_{2}}\left(v_{i}-{\hat{v}}_{i}\right)}^{2}}.$$

Here, *v_i_* is the natural logarithm of the *i*th observed (scanned) petal area, $\hat{v}$*_i_* is the natural logarithm of the *i*-th predicted petal area using the ME, and *n*_2_ represents the number of petals.

We used two models to describe the relationship between *A*_T_ and two one-dimensional measures, *L*_KS_ and *W*_KS_ (Table 1). The Montgomery–Koyama–Smith equation (MKSE) hypothesizes that *A*_T_ scales isometrically with a rectangle, with *L*_KS_ and *W*_KS_ as its two sides (i.e., *A*_T_ ∝ *L*_KS_*W*_KS_). The power-law equation (PLE) reflects the proportionality between *A*_T_ (based on planar projection) and the rectangle bordered by *L*_KS_ and *W*_KS_ [i.e., *A*_T_ ∝ (*L*_KS_*W*_KS_)^α^, where α is the scaling exponent], as many biological measures exhibit a power-law relationship [35]. During data fitting, we performed logarithmic transformation on the observations of *A*_T_ to stabilize the variance (Table 1). Linear fitting was performed using ordinary least-squares to estimate the parameters.

The RMSE and the Akaike information criterion (AIC) were used to measure the goodness of fit and the trade-off between the goodness of fit and model’s structural complexity for MKSE and PLE. The RMSE is calculated as follows:

(5) $$\mathrm{RMSE}=\sqrt{\frac{\sum_{b=1}^{n}{\left(y_{b}-{\hat{y}}_{b}\right)}^{2}}{n}},$$

where *y_b_* is the natural logarithm of the observed *A*_T_ of the *b-*th flower, and $\hat{y}$*_b_* is the natural logarithm of the predicted *A*_T_ of the *b*th flower. The AIC [36] is calculated as follows:

(6) $$\mathrm{AIC}=2p-2\log\left(L\right),$$

where *p* represents the number of model parameter(s) plus 1 for the error term, and log(*L*) represents the maximum log-likelihood of the estimated model [37]. The smaller the RMSE and AIC values, the better the model’s goodness of fit. In addition, the percentage error (PE in %) was used to determine whether it is worthwhile to introduce an additional parameter to the simpler model structure:

(7) $$\mathrm{PE}=\frac{{\mathrm{RMSE}}_{1}-{\mathrm{RMSE}}_{2}}{{\mathrm{RMSE}}_{1}}\times 100\%.$$

Here, the subscripts 1 and 2 represent the MKSE and PLE listed in Table 1. The introduction of an additional parameter can enhance the goodness of fit, which is reflected as a smaller RMSE. The PLE has one additional parameter compared to the MKSE, so RMSE_2_ ≤ RMSE_1_. Empirically, PE > 5% indicates that it is worthwhile to add an additional parameter; otherwise, it is preferable to use a simpler model [17,38].

All analyses were performed using R software.

## 3. Results

**Figure 2:**
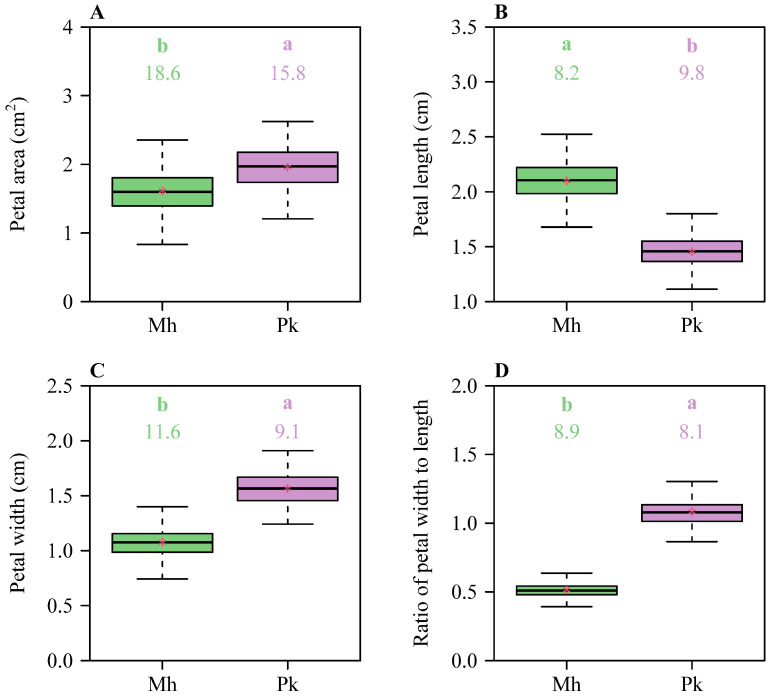
Boxplots of (**A**) petal area, (**B**) petal length, (**C**) petal width, and (**D**) the ratio of petal width to length for the two Rosaceae species. The upper and lower borders of each box represent the 3/4 and 1/4 quantiles, respectively. Significant differences between the species are indicated by the colorful letters a and b in each panel, based on the HSD test (α = 0.05). The horizontal bold lines in the boxes represent the medians, and the asterisks represent the means. Mh represents *M. halliana* var. *Parkmanii*, and Pk represents *P. × kanzakura* cv. *Kawazu-zakura*.

**Figure 3:**
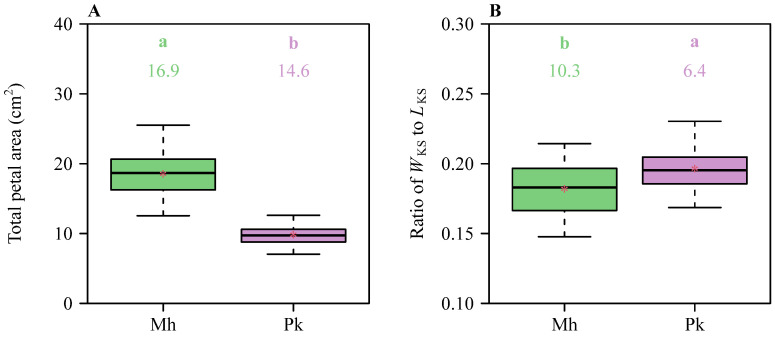
Boxplots of (**A**) the total petal area per flower and (**B**) the ratio of the maximum petal length to the sum of the petal widths per flower for the two Rosaceae species. The upper and lower borders of each box represent the 3/4 and 1/4 quantiles, respectively. Significant differences between the species are indicated by the colorful letters a and b in each panel, based on the HSD test (α = 0.05). The horizontal bold lines in the boxes represent the medians, and the asterisks represent the means. Mh represents *M. halliana* var. *Parkmanii*, and Pk represents *P. × kanzakura* cv. *Kawazu-zakura*.

There were significant differences in petal area (*A*), petal length (*L*), petal width (*W*), and the ratio of petal width to length (*W*/*L*) between the two Rosaceae species (Figure 2), indicating significant variations in petal size and shape. The means of *A*, *W*, and *W*/*L* of *P. × kanzakura* cv. *Kawazu-zakura* (Pk) were significantly greater than those of *M. halliana* var. *Parkmanii* (Mh), while the mean *L* of Mh was significantly greater than that of Pk. This indicates that Pk has larger petals than Mh, with Pk’s petals being shorter and wider, while those of Mh are longer and narrower. There were significant differences in the total petal area (*A*_T_) and the ratio of *W*_KS_ to *L*_KS_ (*W*_KS_/*L*_KS_) between the two species (Figure 3), with the mean *A*_T_ of Mh being significantly greater than that of Pk, while the mean *W*_KS_/*L*_KS_ of Pk was slightly greater than that of Mh. This indicates that Mh has a significantly greater total petal area per flower compared to Pk, while Pk exhibits a significantly higher ratio of maximum petal length to the sum of petal widths per flower than Mh.

**Figure 4:**
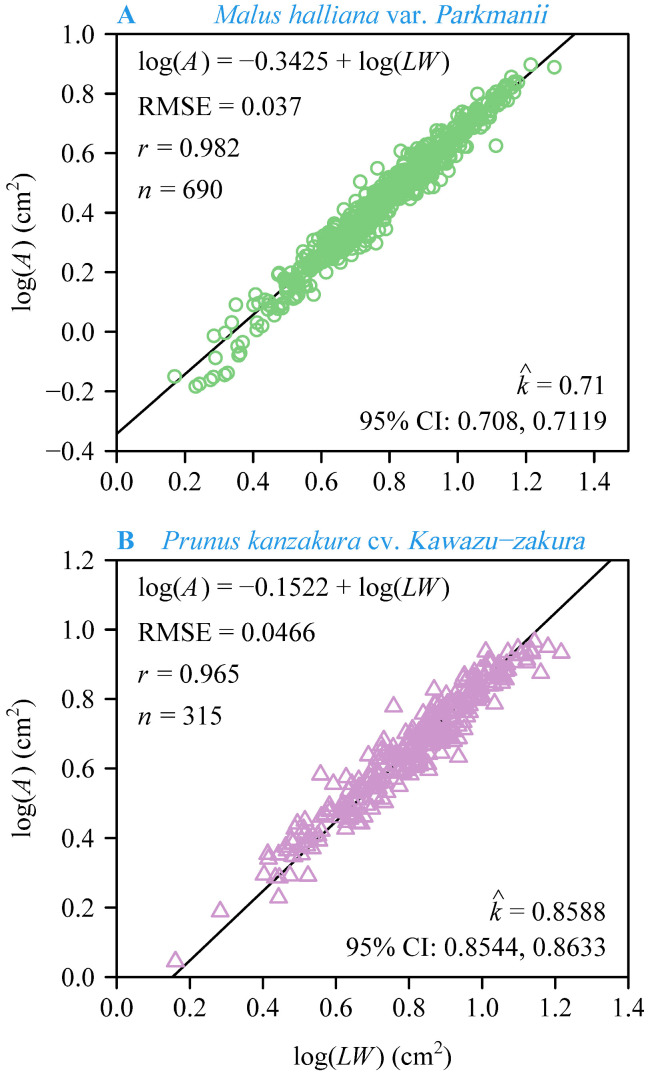
Results of fitting the Montgomery equation on a log–log scale for *M. halliana* var. *Parkmanii* (**A**), and *P. × kanzakura* cv. *Kawazu-zakura* (**B**). In each panel, *A*, *L*, and *W* are the petal area, length, and width, respectively; RMSE is the root-mean-square error; *n* is the total number of petals for each species; $\hat{k}$ is the estimated Montgomery parameter; 95% CIs are the 95% confidence intervals of the Montgomery parameter based on 3000 bootstrap repetitions.

There was a significant proportional log–log relationship between *A* and *LW* for both species (Figure 4). The estimated value of the Montgomery parameter for Mh (0.71) was smaller than that for Pk (0.8588). The RMSE values of the linear regression were 0.0370 for Mh and 0.0466 for Pk, which verified the validity of the Montgomery equation in describing the relationship between *A* and *LW* for Mh and Pk.

**Figure 5:**
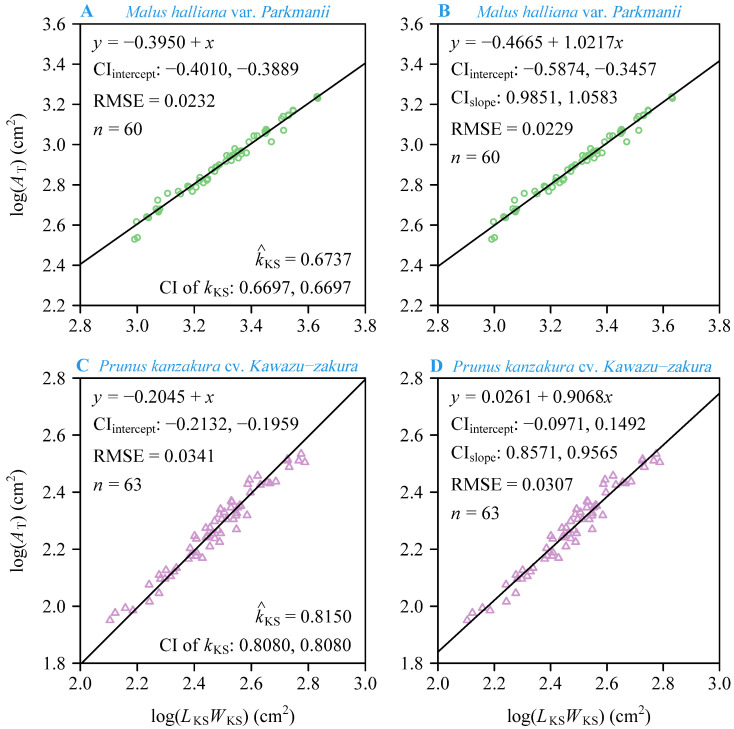
Results of fitting the Montgomery–Koyama–Smith equation and the power-law equation for the relationship between the total petal area per flower (*A*_T_) and the product of the sum of petal widths and the maximum petal length per flower (*L*_KS_*W*_KS_) on a log–log scale for *M. halliana* var. *Parkmanii* (**A**,**B**) and *P. × kanzakura* cv. *Kawazu-zakura* (**C**,**D**). The icons represent the observations converted on the log–log axis; CI_intercept_ is the 95% confidence interval of the intercept; CI_slope_ is the 95% confidence interval of the slope; RMSE is the root-mean-square error of the linear fitting; and *n* is the number of flowers for each species. In panels (**A**,**C**), $\hat{k}$_KS_ is the estimate of the proportionality coefficient of the MKSE, and the CI of $k_{KS}$ represents the 95% confidence interval of the proportionality coefficient.

The MKSE and PLE effectively described the relationship between *A*_T_ and *L*_KS_*W*_KS_ for both species, as indicated by RMSE values below 0.05 (Table 2). Therefore, we further plotted the fitting results of the MKSE and PLE for both species. The RMSE values of the PLE for the two species were slightly smaller than those of the MKSE. This indicates that the goodness of the PLE was better than that of the MKSE for both species. Based on the AIC values, the MKSE had a lower AIC than the PLE for Mh, whereas the PLE had a lower AIC than the MKSE for Pk (Table 2). This indicates that for these two species, the MKSE and PLE represent the matching models suitable for them. At the species level, the estimated value of the proportionality coefficient of the MKSE for Mh (0.6737) was smaller than that for Pk (0.8150) (Figure 5). Additionally, the PE between the two equations was < 5% for Mh, whereas for Pk, it was 9.87%, which exceeded 5%. This indicates that for Mh, it is preferable to use the MKSE as it is a simpler model, whereas for Pk, it is worthwhile to use the PLE despite its greater complexity, as it adds an additional parameter compared to the MKSE. The 95% confidence intervals (CIs) of the slope of the PLE of Mh included unity (Figure 5B), while the upper limit of the 95% CIs of the slope of the PLE of Pk was smaller than unity (Figure 5D). This means that increases in *A*_T_ of Mh keep pace with increases in *L*_KS_*W*_KS_, whereas increases in *A*_T_ of Pk do not keep pace with increases in *L*_KS_*W*_KS_.

**Figure 6:**
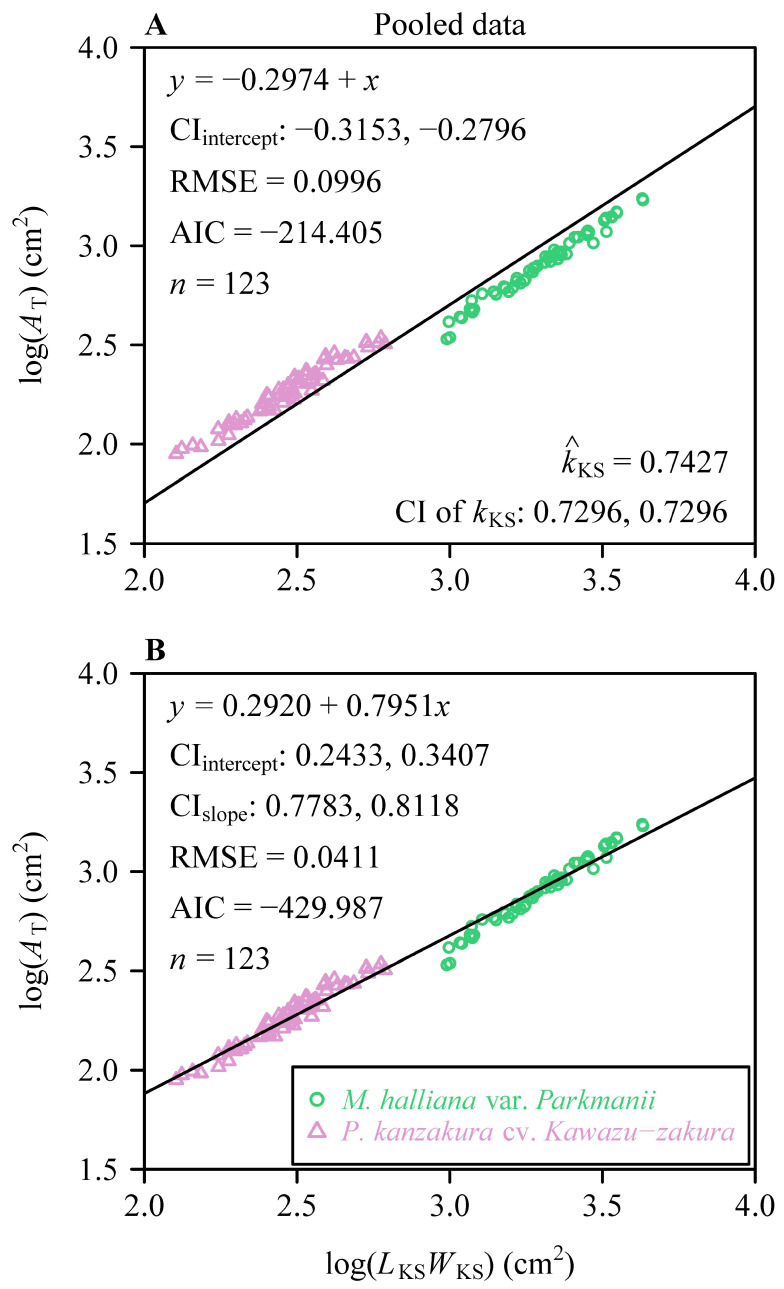
Results of fitting the Montgomery–Koyama–Smith equation (**A**) and the power-law equation (**B**) for the relationship between the total petal area per flower (*A*_T_) and the product of the sum of petal widths and the maximum petal length per flower (*L*_KS_*W*_KS_) on a log–log scale for the pooled data of the two Rosaceae species. The icons are the observations converted on the log–log axis; CI_intercept_ is the 95% confidence interval of the intercept; CI_slope_ is the 95% confidence interval of the slope; RMSE is the root-mean-square error of the linear fitting; and *n* is the total number of flowers of the two species. In panel (**A**), $\hat{k}$_KS_ represents the estimate of the proportionality coefficient of the MKSE, and the CI of $k_{KS}$ represents the 95% confidence interval of the proportionality coefficient.

At the combination data level, the pooled data show that the estimated value of the proportionality coefficient of the MKSE was 0.7427, and the RMSE of MKSE fitting the product of *L*_KS_ and *W*_KS_ to predict *A*_T_ for the two species was 0.0996, while the value of AIC was −214.405 (Figure 6A). The upper limit of the 95% confidence intervals (CIs) for the slope of the power-law equation fitting the two species was smaller than unity, and the RMSE of the power-law equation fitting the product of *L*_KS_ and *W*_KS_ to predict *A*_T_ for the two species was 0.0411, while the value of AIC was −429.978 (Figure 6B).

## 4. Discussion

This study focused on two Rosaceae species to test the relationship between petal area and the product of petal length and width, and the relationship between the total petal area per flower (*A*_T_) and the product of the sum of petal widths and the maximum petal length per flower (*L*_KS_*W*_KS_). The following three sections discuss the ecological significance of the differences in petal traits between the two species; the applicability of the Montgomery equation (ME) for predicting petals with more complex shapes and the reasons for the differences in the Montgomery parameters (MPs), and the selection of a more appropriate model for predicting *A*_T_.

### 4.1. Comparison of Petal Area, Length, Width, and the Ratio of Petal Width to Length Between the Two Species

Petal area (*A*), length (*L*), and width (*W*) were used to quantify petal size, and the ratio of petal width to length (*W*/*L*) was used to quantify petal shape [23]. In terms of petal size, the mean *A* of *P. × kanzakura* cv. *Kawazu-zakura* (Pk) is greater than that of *M*. *halliana* var. *Parkmanii* (Mh). The petals of Pk are broadly ovate to rounded with a concave apex, while the petals of Mh are relatively regular (Figure 1). Therefore, visually, the petals of Pk are more complex in morphology. In nature, the size and morphology of flowers in individual plants vary greatly within species and even within populations [39,40,41,42]. The evolution of floral organ size and dimensions is often closely linked to the probability of pollination success and the need to ensure successful plant reproduction [43]. Additionally, pollinator visitation, as well as the regulation of genetic and environmental factors, also influences the evolution of floral organ size and shape [44,45]. The increase in corolla size is usually closely related to attracting pollinators [46]. Generally, pollinators tend to favor flowers that are larger and more conspicuous [47,48,49]. For example, both bees and flies prefer wild radish (*Raphanus raphanistrum*) flowers with larger sizes [50]. Plant–pollinator interactions are closely related to the evolution of flower symmetry [51,52]. Generally, flower symmetry has two main types: radial symmetry (representing an ancestral state) and bilateral symmetry (representing a derived state) [51,53]. Compared to radially symmetrical flowers, bilaterally symmetrical flowers typically rely more on specialized pollination and exhibit larger sizes, more showy shapes, and restricted nectaries to help attract more selective species of pollinators [54]. Pk blooms in early spring, a time when pollinator activity is lower, whereas Mh blooms during periods of relatively higher pollinator activity. The flowers of Pk display bilateral symmetry, while the flowers of Mh exhibit radial symmetry. Therefore, the corolla of Pk requires larger sizes and more showy shapes to attract these less active and selective pollinators.

The mean *W*/*L* of Pk is greater than that of Mh. In terms of petal shape, the petals of Mh are longer and narrower, whereas the petals of Pk are shorter and wider (Figure 1). The corolla of plants not only protects the delicate reproductive parts inside but also provides a landing platform for pollinators [8,9,10]. Therefore, Mh has evolved numerous obovate petals to maximize the use of available space, while Pk has evolved oval-shaped petals, which only require one round of growth to effectively utilize the space. The differences in petal shape between these two species may reflect the trade-off between the number of petals and petal shape, as well as the reproductive strategies of the plants. The total petal area per flower (i.e., corolla area) of Mh is larger than that of Pk, probably because of the fact that Mh has a greater number of petals than Pk. The flowers of Mh exhibit radial symmetry and are present during periods when pollinators are more active, attracting many unrelated pollinators [54,55]. Therefore, its flowers require a greater number of petals to support these pollinators. In contrast, the flowers of Pk display bilateral symmetry and are present during periods when pollinators are less active, leading to a preference for larger, more attractive shapes and fewer petals to highlight bilateral symmetry. On the other hand, the mean ratio of *W*_KS_ to *L*_KS_ (*W*_KS_/*L*_KS_) of Pk is larger than that of Mh. At the same time, the proportionality coefficient of Pk (0.8150), predicted by the MKSE, is larger than that of Mh (0.6737), which is similar to the prediction of the single petals of the two species by the ME. In other words, the larger *W*/*L* or *W*_KS_/*L*_KS_, the greater the proportionality coefficient predicted by the models.

### 4.2. Calculating Petal Area Using the Montgomery Equation

The Montgomery equation (ME) has been proven effective in predicting leaf area for various simple and complex leaf shapes, including lanceolate, elliptic, ovate, orbicular, cordate, deltoid, hastate, sagittate, and sectorial leaves [19,20,21,22]. In addition, Zhang et al. [56] validated the effectiveness of the ME in predicting stomatal area in four species of Magnoliaceae, and Wang et al. [23] demonstrated that the equation is also effective for predicting the tepal area of *M.* × *soulangeana*. In summary, the ME has been established as a reliable method for estimating the 2D area of leaves and other organic structures.

**Figure 7:**
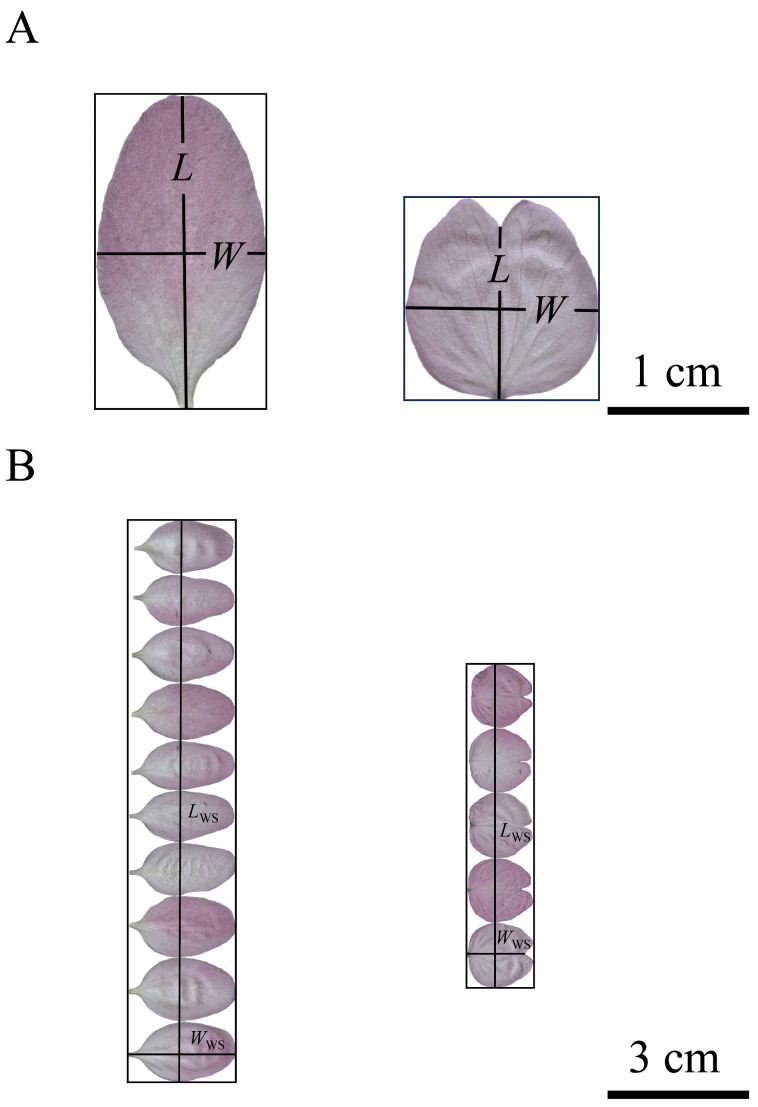
A diagram illustrating the fitting of petal area (*A*) to the product of length (*L*) and width (*W*) using the Montgomery equation, and the fitting of corolla area to the product of length (*L*_KS_) and width (*W*_KS_) using the Montgomery–Koyama–Smith equation, for the two Rosaceae species, which hypothesizes that (*A*) *A*_T_ scales isometrically with a rectangle, with (*L*) *L*_KS_ and (*W*) *W*_KS_ as its sides. In subfigures (**A**, **B**), the left panel displays *M. halliana* var. *Parkmanii*, and the right panel displays *P. × kanzakura* cv. *Kawazu-zakura*.

The concept of serial homology emerged in J. W. von Goethe’s writings in the 18th century, and the classical botanical theory interprets floral parts as metameric homologues of foliage leaves, a view that has since been confirmed by genetic studies [1,2]. Therefore, the ME is expected to be used for predicting petal area. At the species level, the results showed a significant log–log proportionality between the petal area and the product of petal length and width for both species, with high correlation coefficients (*r* = 0.982 for Mh and *r* = 0.965 for Pk) and low RMSEs (RMSE = 0.037 for Mh and RMSE = 0.0466 for Pk) (Figure 4). This suggests that our results are statistically robust. By comparing the fit of the ME to the petal area and the product of petal length and width for the two species (i.e., Mh and Pk), we found that the ME fits better for Mh, which has a simpler petal shape. However, this could also be related to the sample size, as the total number of petals for Pk is *n* = 315, whereas the total number for Mh is *n* = 690. Nonetheless, in terms of the overall fit, the ME is effective for both types of petals. Additionally, the numerical value of the MP for Mh is 0.71. This falls in the range between 1/2 and π/4 of the MP for leaves [17,19,20,21]. However, the predicted MP for Pk is 0.8588, which exceeds the range of 1/2 to π/4. This difference may be due to the significant variation in petal shape between the two species. The petals of Mh are obovate, which are similar to many obovate leaves, whereas the petals of Pk are broadly ovate to rounded with a concave apex (see Figure 1). The ME assumes that area is proportional to length and width, i.e., *A* = *k* × *LW*, where *L* represents both the length of the petal and the length of the rectangle, while *W* represents both the width of the petal and the width of the rectangle. However, due to the concave apex of the petals of Pk, the length that we defined is shorter than the length of the actual rectangle, resulting in a larger MP (see Figure 7). In addition, the applicability of the ME may be reduced for species with more complex petal shapes, so it is expected that more complex models will be developed in the future to predict their petal area more accurately.

### 4.3. Comparison of the Montgomery–Koyama–Smith Equation (MKSE) and the Power-Law Equation (PLE) for Estimating the Total Petal Area per Flower

By comparing the RMSE and AIC values, the MKSE and the PLE can both be shown to well describe the relationship between *A*_T_ and *L*_KS_*W*_KS_ (Table 2). The MKSE is used to estimate the total leaf area per shoot by using the collective dimensions of the leaves on a shoot, and it has been shown to be effective in predicting the total leaf area for a wide range of diverse taxa [26,27]. The PLE introduces a scaling exponent based on the MKSE, which improves the goodness of fit.

At the species level, the data show that both the MKSE and the PLE are valid for estimating the corolla area of Mh, as evidenced by the low RMSE and AIC values. Additionally, the percent error between the two models was <5%, suggesting that the MKSE is better than the more complex PLE for calculating the corolla area of Mh. By contrast, for calculating the corolla area of Pk, the PLE fits better. This is evidenced by the fact that the RMSE and AIC values fitted by the PLE were smaller than those of the MKSE, and the percent error between the two models for Pk was equal to 9.87%. In addition, the results show that the 95% confidence interval of the slope of the PLE fitted to Mh’s *A*_T_ and *L*_KS_*W*_KS_ included unity, indicating a strong proportional relationship between its *A*_T_ and *L*_KS_*W*_KS_. This implies that the simpler structure of the MKSE is more suitable for predicting Mh’s *A*_T_. In contrast, the upper limit of the 95% confidence interval of the slope of the PLE fitted to Pk’s *A*_T_ and *L*_KS_*W*_KS_ was less than unity, suggesting that Pk’s petal shapes are more complex, and therefore, the use of the PLE is more appropriate. For the pooled data, the goodness of fit of the PLE was significantly better than that of the MKSE, as evidenced by lower RMSE and AIC values (Figure 6). However, the petal shapes of the two species we studied were distinctly different; the petals of Mh were obovate, while the petals of Pk were broadly ovate to rounded with a concave apex (Figure 1). Therefore, for flowers with simple petal shapes, such as Mh, the MKSE can be used to predict the corolla area, while for flowers with complex petal shapes, like Pk, we suggest using the PLE, which has a more complex model structure, to predict the corolla area. Meanwhile, future sampling will need to be expanded to determine which model is better for predicting the corolla area within the Rosaceae family.

## 5. Conclusions

In this study, we analyzed petal traits in two Rosaceae species and verified the validity of the Montgomery equation (ME) for describing the relationship between the individual petal area and the product of petal length and width, as well as the validity of the Montgomery–Koyama–Smith equation (MKSE) for estimating the total petal area per flower (i.e., corolla area). The study of petal traits in the two Rosaceae species provides important insights for understanding plant reproductive strategies. Furthermore, the results show that the ME is effective in estimating petal area for both Rosaceae species. For *M*. *halliana* var. *Parkmanii*, the MKSE is more suitable than the power-law equation (PLE), because it has a better goodness of fit and a simpler model structure with only one parameter compared to the PLE. However, the PLE is more effective in estimating the corolla area of *P. × kanzakura* cv. *Kawazu-zakura*. The differences in model performance for estimating petal area/corolla area between the two species stem from variations in petal shape and sample size. Furthermore, flower size is closely related to plant reproduction, and the corolla partially reflects the size of the floral organ. Therefore, our study provides a tool for predicting the area of similarly shaped petals or corollas, thereby indirectly supporting research on plant reproductive strategies. To improve efficiency, more optimized models will be needed to predict the petal area of species with more complex petal shapes in the future.

## Figures and Tables

**Table 1:** The two models for calculating the total petal area per flower in this study.

Model	Model Expression	Log Transformation of Model
MKSE	$A_{T}=k_{\mathrm{KS}}\left(L_{\mathrm{KS}}W_{\mathrm{KS}}\right)$	$\log(A_{T})=c+\log\left(L_{\mathrm{KS}}W_{\mathrm{KS}}\right)$
PLE	$A_{T}=\beta {\left(L_{\mathrm{KS}}W_{\mathrm{KS}}\right)}^{\alpha }$	$\log(A_{T})=\gamma +\alpha \log\left(L_{\mathrm{KS}}W_{\mathrm{KS}}\right)$

Here, the Montgomery–Koyama–Smith equation (MKSE) and the power-law equation (PLE) were used. To stabilize the variance of *A*_T_, the log-transformed version of each model was used.

**Table 2:** RMSE and AIC values for the total petal area per flower that were calculated based on the Montgomery-Koyama-Smith equation (MKSE) and the power-law equation (PLE).

Model	*Malus halliana* var. *Parkmanii*		*Prunus kanzakura* cv. *Kawazu-zakura*	
	RMSE	AIC	RMSE	AIC
MKSE	0.0232	−277.5971	0.0341	−242.9052
PLE	0.0229	−277.0368	0.0307	−253.9949

## Data Availability

The data can be found in the online Supplementary Table S1.

## Supplementary Materials

The following supporting information can be downloaded at https://www.mdpi.com/article/10.3390/plants13243499/s1, Table S1: The raw data on petal area, petal length, and petal width of the two Rosaceae species.
